# Assessing perceived participation among older adults total hip arthroplasty and total knee arthroplasty patients six months post-surgery: a cross-sectional study

**DOI:** 10.3389/fpubh.2023.1282461

**Published:** 2023-11-13

**Authors:** Lifeng Yao, Qin Jia, Jiayun Wu, Yufei Chai, Chu Gao, Yingying Wang, Ke Li, Meihong Lai

**Affiliations:** ^1^Hangzhou Hospital of Traditional Chinese Medicine, Hangzhou TCM Hospital Affiliated to Zhejiang Chinese Medical University, Hangzhou, China; ^2^School of Nursing, Zhejiang Chinese Medical University, Hangzhou, China; ^3^Zhejiang Provincial People's Hospital (People's Hospital of Hangzhou Medical College), Hangzhou, China; ^4^Hubei Cancer Hospital, Wuhan, China

**Keywords:** aged, arthroplasty, replacement, participation, rehabilitation, daily living activities, psychological well-being

## Abstract

**Aims:**

This research sought to assess the perceived levels of participation and autonomy in senior patients who had received total hip arthroplasty (THA) or total knee arthroplasty (TKA) in Hangzhou, China. Furthermore, the study aimed to identify the factors linked to these outcomes.

**Study design:**

This investigation will utilize a cross-sectional study design to assess perceived participation and autonomy among older adults total hip arthroplasty (THA) and total knee arthroplasty (TKA) patients. The research was conducted in Hangzhou, China, at a tertiary hospital.

**Methods:**

Convenient sampling was utilized to select 139 patients who underwent THA or TKA between March 2022 and March 2023 and met the inclusion criteria at a tertiary hospital in Hangzhou. The Impact on Participation and Autonomy Questionnaire, Hip/Knee Injury and Osteoarthritis Outcome Score (HOOS/KOOS), 5-Item Geriatric Depression Scale, Multidimensional Scale of Perceived Social Support, and Elders Health Empowerment Scale were used to assess perceived participation, hip/knee-related symptoms and functional restrictions, depression symptoms, social support, and health empowerment.

**Results:**

The mean score for perceived participation and autonomy was 22.554 (SD: 13.042). The mean scores for participation in indoor autonomy, outdoor autonomy, family roles, and social relations were 0.654 (SD: 0.608), 1.324 (SD: 0.792), 1.053 (SD: 0.657), and 0.664 (SD: 0.542), respectively. Negative correlations were observed between perceived participation/autonomy scores and HOOS/KOOS, social support, and health empowerment scores. Conversely, a positive correlation was found between perceived participation/autonomy scores and depression scores. The detrimental effect of HOOS/KOOS, social support, and health empowerment scores on perceived participation and autonomy was notable, while the impact of depressive symptoms was comparatively minor.

**Conclusion:**

Older Chinese patients, at first six months post THA/TKA surgery, reported higher levels of perceived participation compared to individuals with other conditions, such as stroke patients. Functional limitations resulting from hip/knee-related symptoms, as well as social support and health empowerment, emerged as significant influencing factors for perceived participation and autonomy. This research enhances our comprehension of the elements influencing perceived participation among older adults individuals who have undergone THA/TKA procedures.

## 1. Introduction

The frequency of Total Hip Arthroplasty (THA) and Total Knee Arthroplasty (TKA) procedures has surged rapidly, driven by the significant growth of the older adults population in China and other countries. Model forecasts project a staggering 176% increase in THA cases and a 139% rise in TKA cases by the year 2040 (1). However, despite this surge in surgeries, many patients exhibit disappointingly modest levels of postoperative activity. Following the surgical procedures, a substantial 20% of patients experience social isolation, and a range of 7 to 23% encounter persistent long-term discomfort (2). Furthermore, individuals requiring THA or TKA are increasingly concerned about their ability to return to work and actively participate in society (3).

A more complete approach to recovery will involve looking at a lot of different outcome measures that show physical and mental impairments, activity limitations, and participation restrictions, as shown in the International Classification of Functioning (ICF) model at different follow-up points (4).

Therefore, there is a pressing need for research into factors associated with involvement in order to encourage full participation of patients following TKA or THA.

Until now, the focus has predominantly been on assessing hip or knee function and a limited set of activities such as mobility and walking. The majority of studies have primarily examined the connections between participation and rehabilitation outcomes in the assessment of THA or TKA participation (5, 6). Factors influencing patients’ participation have often been investigated using objective indicators, such as different surgical approaches and physiological function (6–8). However, participation after a THA or TKA can be influenced by a number of factors. Sergooris et al. (9) indicated depression, social support, self-efficacy, and other characteristics may be connected to activities and engagement after total hip arthroplasty (THA). Maxwell et al. (10) showed that reduced participation was linked to females, depressed symptoms, and significant knee pain in either knee. Hylkema et al. (11) illustrated that among working-age TKA patients, activity, mental, and physical limitations have a substantial impact on participation constraints. Overall, it is significant to highlight that the evidence for those relationship varies at different periods among different articles. Diverse research have shown inconsistent results.

The long-term effects of psychological and social functioning are crucial for postoperative patients, according to the biopsychosocial model, in addition to the physical recovery (12, 13). Especially in empowerment, Patients who have a high level of health empowerment frequently have greater self-care skills and self-efficacy in rehabilitation (14). Caillouet et al. (15) discovered that health empowerment could encourage positive health behaviors, such as exercise, among community-dwelling older adults individuals.

In conclusion, there is a need for exploratory studies focusing on participation among THA or TKA patients following surgery, with health empowerment, depression, and perceived social support potentially shedding light on participation dynamics. However, the majority of existing research on participation recovery after THA or TKA predominantly centers on Western populations. Hence, the aim of this study is to identify the variables associated with perceived participation among THA or TKA patients at first six months post-surgery in China.

## 2. Methodology

### 2.1. Aim

The main aim of this study was to evaluate the perceived levels of participation and autonomy among Chinese patients who had undergone Total Hip Arthroplasty (THA) or Total Knee Arthroplasty (TKA).

### 2.2. Design

This study employed an exploratory cross-sectional research design.

### 2.3. Participants

Subjects were enlisted from the orthopedic division of a hospital graded as 3A, in accordance with the Hospital Grading Management Measures and pertinent regulations governing medical establishments in mainland China. Eligibility criteria for potential participants were as follows: (a) age 60 years or older, (b) underwent primary THA or TKA surgery, (c) which is at first 6-month had passed since the surgery, (d) capable of completing the surveys in Chinese both physically and cognitively, and (e) provided informed consent to participate in the study after being duly informed and signing the informed consent form. Exclusion criteria encompassed patients who: (a) underwent contralateral TKA or contralateral THA within six months prior to the surgery, (b) had various ailments that impeded walking, (c) were significantly organ dysfunctional or critically ill, and (d) were diagnosed with mental illnesses. Sample size = number of dimensions for main variable * (5–10)/(100%–10%) =14 * (5–10)/(100%–10%) = [78 ~ 156] (16).

### 2.4. Data collection

Data collection was conducted between March 2022 and March 2023 at a tertiary hospital located in Hangzhou. Prior to commencing the investigation, all investigators underwent a comprehensive briefing regarding the study’s inclusion and exclusion criteria, effective communication techniques, and data collection procedures. Following obtaining informed consent, participants were provided with survey instructions. Subsequently, communication was established with the patients through phone calls or WeChat, a widely-used social media and messaging application in China, to gain insights into their condition and arrange outpatient follow-up appointments. Surveys were administered and data was collected from participants through a combination of phone or WeChat communication and in-person visits to the hospital.

This research design, participant recruitment criteria, and data collection methods aimed to comprehensively explore the perceived participation and autonomy levels among Chinese THA and TKA patients, shedding light on their experiences and factors influencing their postoperative outcomes.

### 2.5. Instruments

#### 2.5.1. Impact on participation and autonomy questionnaire (IPA)

The IPA, developed by Dutch researchers Cardol et al. (17), was employed to gauge patients’ perceived participation and autonomy. In this study, the Chinese version of IPA (C-IPA) was utilized to assess patients’ perceived participation levels. C-IPA comprises four dimensions: autonomy indoors (seven items), autonomy outdoors (five items), social life and relations (six items), and family role (seven items). Respondents rate each item on a 5-point Likert scale ranging from 0 (completely consistent) to 4 (completely inconsistent). Higher scores indicate greater levels of perceived participation, generating a total score between 0 and 100 (18). The Cronbach’s alpha values for each domain of IPA-Chinese have been reported to range from 0.81 to 0.91 across diverse demographics, including stroke and burns patients (19, 20).

#### 2.5.2. Hip/knee injury and osteoarthritis outcome score (HOOS/KOOS)

The HOOS and KOOS instruments are self-administered questionnaires employed for evaluating symptoms and functional limitations related to the hip or knee. The HOOS, consisting of 40 items, assesses five patient-centered domains: pain (10 items), symptoms encompassing stiffness and range of motion (5 items), activity limitations in daily living (17 items), sport and recreational function (4 items), and hip-related quality of life (4 items) (21). Similarly, the KOOS consists of five domains: pain (9 items), symptoms (7 items), activities of daily living (17 items), sport and leisure function (5 items), and knee-related quality of life (4 items) (22). The score for each domain is calculated by summing individual item scores, which range from 0 to 4. These subscale scores are then transformed to a scale of 0 (indicating no issues) to 100 (indicating severe problems). Notably, the HOOS has demonstrated strong reliability, as indicated by intra-class correlations ranging from 0.75 to 0.97 and Cronbach’s alpha values of 0.82 to 0.98 (23–26). Each HOOS-Chinese dimension has a Cronbach’s alpha value between 0.865 to 0.968 (27). The Cronbach’s alpha values for each KOOS-Chinese dimension range from 0.80 to 0.94 (28).

#### 2.5.3. 5-item geriatric depression scale (GDS-5)

The GDS-5, derived from the GDS-15, evaluates depressive symptoms in the older adults. Respondents provide scores of 0 or 1 for each of the five items, which are then summed to indicate symptom intensity. Scores of 2 or higher indicate the presence of symptoms, while scores of 0 or 1 suggest their absence (29). GDS-5 has strong test–retest and interrater reliability, with scores of 0.88 and 0.84, respectively (29). The Cronbach’s alpha coefficient of GDS-5 in Chinese was between 0.603–0.61 (30, 31).

#### 2.5.4. Multidimensional scale of perceived social support (MSPSS)

The MSPSS, comprising 12 items, evaluates individuals’ perceptions of their social networks, encompassing family support (items 3, 4, 8, and 11), friend support (items 6, 7, 9, and 12), and significant other support (items 1, 2, 5, and 10). Using a 7-point Likert scale to gauge agreement, higher scores (ranging from 12 to 94) denote stronger perceived social support (32). The Chinese version of the MSPSS has consistently demonstrated reliability across diverse samples (33).

#### 2.5.5. Elders health empowerment scale (EHES)

The EHES, developed by Park and Park (34), is designed to assess the older adults’s capacity to manage health-related matters. The Chinese version of EHES (C-EHES) consists of eight items, representing seven health empowerment facets: control, problem-solving, decision-making, self-motivation, psychosocial coping, resource utilization, and self-efficacy. C-EHES items are rated on a 5-point Likert scale, where higher scores (ranging from 8 to 40) indicate greater levels of health empowerment. The EHES has exhibited a Cronbach’s alpha value of 0.80 (34). The Cronbach’s alpha coefficient of Chinese version of EHES is 0.838 (35).

#### 2.5.6. Demographic and clinical information questionnaire

A custom-designed questionnaire was utilized to gather demographic and clinical information, encompassing gender, age, educational attainment, marital status, place of residence, sources of income, monthly income, type of surgery, existing comorbidities, apprehension about falling, additional complications, and presence of painful joints in other regions.

### 2.6. Ethical considerations

The Ethics Review Board of Zhejiang Provincial People’s Hospital granted approval for this study. Participants were assured that their involvement would have no adverse repercussions for them or their loved ones and that participation was entirely voluntary. All participants provided written consent before participating.

### 2.7. Data analysis

Statistical analysis was performed using SPSS 26.0 software, encompassing descriptive analysis, Spearman/Pearson correlation analysis, and multiple linear regression. Mean and standard deviation (SD) for each IPA domain were calculated. Relationships between perceived participation and quantitative factors (hip/knee-related symptoms and functional restrictions, depressive symptoms, social support, and health empowerment) were assessed through Spearman/Pearson correlation analysis. Correlational variables were incorporated into multiple linear regression analysis.

### 2.8. Validity and reliability

All researchers, nursing major in graduate students, received training on research ethics, participant recruitment, and standard operating procedures. Confidentiality and anonymity were maintained to minimize self-reported bias. Questionnaires were promptly reviewed for completeness after each administration. To ensure accuracy, two researchers independently cross-checked interview data entry. Data were coded for analysis purposes and the data’s consistency was examined.

## 3. Results

### 3.1. Participants’ characteristics

A total of 139 patients participated in the study (see Table 1). With 65.25% more female participants than male participants, there is a sizable gender gap. According to the participants’ age distribution, 48.20% are between the ages of 60 and 69, followed by 42.45% between the ages of 70 and 79, and just 9.35% are 80 or older. Indicating poorer educational attainment, a sizable portion of participants (35.25%) are illiterate and 34.53% have only finished elementary school. The proportion of participants who are single, divorced, or widowed is smaller than the proportion who are married (82.01%) 0.33.09% of participants live in urban regions, compared to 66.91% who live in rural areas. Among the surgical treatments, total knee arthroplasty is slightly more frequent (56.83%) than total hip arthroplasty. The majority of individuals (62.59%) report having a fear of falling, compared to 37.41% who do not.87.05% of patients had no extra issues, compared to 12.95% who did. Fewer people (69.06%) report having one or more additional joint pains, with the remainder reporting none at all.

**Table 1:** Descriptive statistics of the participants’ characteristics.

Name	Item	*N*	Percentage (%)
Gender	Male	49	35.25
	Female	90	64.75
Age	60–69	67	48.20
	70–79	59	42.45
	≥80	13	9.35
Education	Illiterate	49	35.25
	Primary school	48	34.53
	Junior high school	27	19.42
	High school	12	8.63
	College and above	3	2.16
Marital status	Unmarried	4	2.88
	Married	114	82.01
	Divorced	5	3.60
	Widowed	16	11.51
Residence	Rural	93	66.91
	Urban	46	33.09
Economic source	Minimum livelihood guarantee	14	10.07
	Support from other family members	34	24.46
	Income from labor	26	18.71
	Retirement pension	65	46.76
Monthly income	≤500	25	17.99
	501–1,000	18	12.95
	1,001–1,500	12	8.63
	1,501–2000	23	16.55
	≥2000	61	43.88
Type of surgery	Total knee arthroplasty	79	56.83
	Total hip arthroplasty	60	43.17
Number of comorbidities	0	51	36.69
	1	57	41.01
	2–3	28	20.14
	>3	3	2.16
Fear of falling	Yes	87	62.59
	No	52	37.41
Presence of other complications	Yes	18	12.95
	No	121	87.05
Number of other joint pains	0	96	69.06
	1	32	23.02
	2–3	11	7.91
Total		139	100.0

Provides descriptive statistics for participants’ characteristics, including gender, age, education, marital status, residence, economic source, monthly income, type of surgery, comorbidities, fear of falling, presence of other complications, and number of other joint pains.

### 3.2. Descriptive data in older adults THA & TKA patients

Table 2 descriptive statistics are presented in the format “Mean Standard Deviation (Min, Max),” with corresponding variable descriptions. The mean score for perceived participation and autonomy was 22.554 (SD: 13.042), in contrasting their mean results, the highest one is participation in autonomy outdoors followed by participation in family role, participation in social relations, and participation in autonomy indoors, representing a ranking of involvement level in reverse. The average HOOS/KOOS score was 390.052 (SD: 62.962), while depression symptom scores averaged 0.583 (SD: 1.221). Social support received a mean score of 58.165 (SD: 8.984), and health empowerment yielded a mean score of 28.849 (SD: 5.595).

**Table 2:** Descriptive statistics of the four domains of the IPA and different variables.

Variable	Item	Mean ± Standard deviation (min, max)	Item mean ± standard deviation
Participation in autonomy indoors	7	4.576 ± 4.253 (0.000, 25.000)	0.654 ± 0.608
Participation in autonomy outdoors	5	6.619 ± 3.962 (0.000, 20.000)	1.324 ± 0.792
Participation in family role	7	7.374 ± 4.602 (0.000, 21.000)	1.053 ± 0.657
Participation in social relations	6	3.986 ± 3.253 (0.000, 18.000)	0.664 ± 0.542
Perceived participation and autonomy	25	22.554 ± 13.042 (1.000, 61.000)	/
HOOS/KOOS	40/42	390.052 ± 62.962 (126.681, 488.889)	/
Depression symptoms	5	0.583 ± 1.221 (0.000, 5.000)	/
Social support	12	58.165 ± 8.984 (26.000, 80.000)	/
Health empowerment	8	28.849 ± 5.595 (14.000, 40.000)	/

Presents descriptive statistics for various variables and the four domains of the IPA (participation in autonomy indoors, Participation in autonomy outdoors, Participation in family role, Participation in social relations), including their respective item means and standard deviations. Additionally, it provides statistics for perceived participation and autonomy, HOOS/KOOS scores, depression symptoms, social support, and health empowerment.

### 3.3. Correlation between perceived participation and different variables

Table 3 outlines the correlations between IPA scores and various factors. Noteworthy IPA-significant correlations were observed with factors including HOOS/KOOS (*r* = −0.587, *p* < 0.01). Depression symptoms (*r* = 0.426, *p* < 0.01), Social support (*r* = −0.477, *p* < 0.01), and Health empowerment (*r* = −0.565, *p* < 0.01).

**Table 3:** Correlation between the perceived participation and HOOS/KOOS, depression systems, social support, and health empowerment.

	IPA
**HOOS/KOOS**	−0.587**
Pain	−0.488**
Symptoms such as stiffness and Range of motion	−0.442**
Activity limitations-ADL	−0.496**
Sport and recreational function	−0.622**
Quality of life-QOL	−0.347**
**Depression symptoms**	0.426**
**Social support**	−0.477**
Family support	−0.294**
Friends support	−0.516**
Significant others support	−0.464**
**Health empowerment**	−0.565**

** Correlation is significant at 0.01 level.

### 3.4. Predictors of perceived participation among older adults THA & TKA patients six months after surgery

Table 4 predictors of perceived participation were analyzed through multiple linear regression. Notable predictors included HOOS/KOOS (*β* = −0.446, *p* < 0.01), Social support (*β* = −0.217, *p* = 0.002), and Health empowerment (*β* = −0.266, *p* < 0.01).

**Table 4:** Multiple linear regression of a set of independent variables related to IPA.

Variable	Unstandardized Coefficient		Standardized Coefficient	*t*	*p*
	*B*	SE	Beta		
Constant	94.374	8.357	-	11.293	<0.001
HOOS/KOOS	−0.092	0.015	−0.446	−6.321	<0.001
Depression symptoms	0.669	0.832	0.063	0.804	0.423
Social support	−0.315	0.102	−0.217	−3.087	0.002
Health empowerment	−0.620	0.153	−0.266	−4.039	<0.001

Displays the results of a multiple linear regression analysis, indicating that perceived participation and autonomy (IPA) are significantly influenced by HOOS/KOOS scores, social support, and health empowerment, while depression symptoms do not have a significant impact.

The study’s results reveal significant associations and predictors of perceived participation and autonomy among older adults THA & TKA patients, offering insights into the factors influencing their postoperative experiences.

## 4. Discussion

Following THA & TKA, an examination of perceived participation was conducted, encompassing its levels and potential determinants.

The average score for perceived participation and autonomy was lower than the findings in Chen et al.’s study (20).

Six months post-surgery, patients in our study demonstrated improved perceived participation compared to research outcomes pertaining to other conditions. Within our study, THA & TKA patients reported higher levels of perceived participation in social interactions and indoor autonomy in comparison to their reported participation in family roles and outdoor autonomy. This discrepancy can be attributed to a combination of factors. On one hand, the older adults patients who underwent hip and knee joint replacement in this study may be more socially engaged due to their enhanced self-esteem and the fulfillment of their desire for respect through positive interactions with family, neighbors, and friends. On the other hand, limitations in joint flexibility and stiffness resulting from hip and knee joint replacement surgery, particularly among older patients, could affect their ability to engage in outdoor activities. Concerns related to safety and the fear of falling might lead patients to prioritize lower-intensity activities that align with their sense of self-identity and value, such as childcare and housekeeping. Consequently, their participation in outdoor activities might decrease (36).

Healthy providers require a reliable resource to identify factors indicative of participation following THA & TKA, aiding in the efficient assessment of high-risk patients with limited participation and guiding care provision. As anticipated, the occurrence of symptoms, functional limitations and Infectious complication associated with the hip and knee stood out as strong indicators of participation (11, 37–38). Therefore, in the context of fostering participation, it is crucial to focus on the early restoration of hip/knee-related symptoms, functional limitations and infectious prevention post-THA & TKA surgery. Orthopedic nursing should encompass teaching THA & TKA-related rehabilitation techniques to facilitate swift and sustained recovery. Besides, the use of prophylactic antibiotics prior to surgery, the application of the asepsis principle throughout the procedure, and the symptoms of postoperative infection should all be thoroughly monitored. It is also important to acknowledge that partial two-stage revision procedures have a beneficial effect on persistent joint replacement infections (38).

Health empowerment is another determinant of patients’ degree of perceived participation and autonomy.

Aligning with findings from studies involving older adults individuals engaged in physical activity within community settings (15). An exploration of causative factors suggests that older adults patients who have undergone hip and knee joint replacement surgery experience an increased motivation for self-health management and participation six months after achieving substantial recovery of physical function and structure. This enhanced motivation propels greater engagement in physical activity, subsequently boosting social interaction levels (15, 39).

Therefore, healthcare providers can learn more about their patients’ post-operative experiences by using qualitative research techniques. Mindfulness treatment and psychological simulation training (including motor imagery and motor observation) can be effectively administered after surgery with the help of honest patient feedback (40, 41). The patient’s sense of agency and ability to care for themselves may increase as a result.

Our conclusion regarding the relationship between social support and engagement resonates with existing research, encompassing studies involving stroke patients (42) and other populations (43). This relationship could stem from the material and emotional support provided by family, friends, and relatives, satisfying patients’ emotional needs and bolstering treatment adherence. Consequently, their confidence in social interaction is heightened (43). As licensed healthcare professionals, nurses play a pivotal role in offering patients nursing care and education, while acting as a crucial link to medical practitioners such as surgeons, physical therapists, home healthcare providers, and social services (44). Therefore, nurses must comprehend how social support from diverse sources impacts the level of social engagement among older adults patients who have undergone hip and knee joint replacement surgery (42, 43). However, the potential impact of host-related factors in these situations must be taken into account. Doctors must unavoidably tell patients to the fullest extent practicable about all relevant risks and potential benefits associated with the procedure, as well as any potential challenges in the management of postoperative recovery. The choice of joint materials and the possibility of postoperative joint wear are also included in this (45).

Additionally, healthy providers can perform proper clinical management and risk stratification to prevent postoperative infection, which can also help to minimize medical litigation (37). Furthermore, nurses can enhance health education guidance for family members, elevating disease and recovery awareness among older patients’ families and caregivers, thereby strengthening internal family support. Consequently, they can better manage the condition collectively, ultimately boosting patients’ confidence in social interaction. Alongside knowledge about the social support system, nurses can also furnish accessible information and educational materials regarding social services. They can effectively harness community resources while actively organizing community events. By guiding patients towards senior activity centers or local parks, nurses can further harness social support and enhance social contact and communication (39). Notably, depression symptoms were not observed to impact patients’ participation levels six months post-surgery in this investigation. This might be attributed to the fact that individuals with pre-existing depression who underwent joint replacement surgery reported diminished depressive symptoms. This improvement could be attributed to the surgery-induced enhancement in joint function and reduction of unfavorable symptoms such as pain (11, 46).

Furthermore, this study included remote follow-up for patients who had joint replacements. In addition to ensuring continuity of treatment, remote healthcare offers a fresh approach to addressing patients’ post-operative recovery demands and consequently raising their post-operative satisfaction. Therefore, in the future, it will be crucial to integrate and foster synergy among financial, organisational, human, and technological resources through national or regional digital health initiatives, under well-structured strategic leadership (47). To ensure the applicability and efficacy of remote healthcare, special emphasis should be given to the creation of pertinent rules and regulations and adherence to ethical standards (48).

Limitations of this study include a sample drawn from a single center, potential subjective biases due to assistance needed from family members for data collection, and a focus on a less educated older adults population. Future research could explore perceived participation among patients with different joint replacements through a stratified approach and develop detailed relationship descriptions using trajectory models.

## 5. Conclusion

Following THA & TKA, participation and autonomy encompass intricate concepts influenced by multiple factors. The recuperation of hip/knee-related symptoms, functional limitations, social support, and health empowerment assumes pivotal significance. In nursing practice, heightened focus should be placed on older patients vulnerable to reduced engagement. By addressing patient concerns through interventions like health empowerment, bolstering social support, and imparting comprehensive hip and knee knowledge through health education, nurses can play a more proactive role in advancing the rehabilitation of THA and TKA among the older adults.

## Data availability statement

The raw data supporting the conclusions of this article will be made available by the authors, without undue reservation.

## Ethics statement

The studies involving humans were approved by Department of Orthopedics, Zhejiang Provincial People’s Hospital. The studies were conducted in accordance with the local legislation and institutional requirements. Written informed consent for participation in this study was provided by the participants’ legal guardians/next of kin.

## Author contributions

LY: Conceptualization, Data curation, Formal analysis, Investigation, Methodology, Software, Writing – original draft, Writing – review & editing. QJ: Supervision, Writing – review & editing. JW: Data curation, Writing – original draft. YC: Conceptualization, Software, Writing – original draft. CG: Conceptualization, Methodology, Writing – original draft. YW: Conceptualization, Investigation, Writing – review & editing. KL: Validation, Writing – review & editing. ML: Formal analysis, Data curation, Writing – original draft.
